# Isolation and characterization of exopolysaccharide with immunomodulatory activity from fermentation broth of *Morchella conica*

**DOI:** 10.1186/2008-2231-21-5

**Published:** 2013-01-05

**Authors:** Chao-an Su, Xiao-yan Xu, De-yun Liu, Ming Wu, Fan-qing Zeng, Meng-yao Zeng, Wei Wei, Nan Jiang, Xia Luo

**Affiliations:** 1 Lishui Agricultural Academy of Sciences, Lishui, Zhejiang, 32300, China; 2 Laboratory of Cellular and Molecular Biology, Sichuan Academy of Chinese Medicine Science, Chengdu 610041, China

**Keywords:** Morchella conica, Exopolysaccharides, Submerged liquid culture, Immunomodulatory activity

## Abstract

**Background and the purpose of this study:**

Mushroom polysaccharides have traditionally been used for the prevention and treatment of a multitude of disorders like infectious illnesses, cancers and various autoimmune diseases. *In vitro and in vivo* studies suggest that certain polysaccharides affect immune system function. *Morchella conica* (*M. conica*) is a species of rare edible mushroom whose multiple medicinal functions have been proven. Thus, the objective of this study is to isolate and characterize of exopolysaccharide from submerged mycelial culture of *M. conica,* and to evaluate its immunomodulatory activity.

**Methods:**

A water-soluble *Morchella conica* Polysaccharides (MCP) were extracted and isolated from the fermentation broth of *M. conica* through a combination of DEAE-cellulose and Sephacryl S-300 HR chromatograph. NMR and IR spectroscopy has played a developing role in identification of polysaccharide with different structure and composition from fungal and plant sources, as well as complex glycosaminoglycans of animal origin. Thus, NMR and IR spectroscopy were used to analyze the chemical structure and composition of the isolated polysaccharide. Moreover, the polysaccharide was tested for its immunomodulatory activity at different concentrations using *in vitro* model.

**Results:**

The results showed that MCP may significantly modulate nitric oxide production in macrophages, and promote splenocytes proliferation. Analysis from HPLC, infrared spectra and nuclear magnetic resonance spectroscopy showed that MCP was a homogeneous mannan with an average molecular weight of approximately 81.2 kDa. The glycosidic bond links is →6)-α-D-Man *p*-(1→.

**Conclusion:**

The results suggested that the extracted MCP may modulate nitric oxide production in macrophages and promote splenocytes proliferation, and it may act as a potent immunomodulatory agent.

## Introduction

Medicinal mushrooms have become an attractive option for functional food or as a source for the development of pharmaceuticals and nutraceuticals. The medicinal properties are due to various cellular components and secondary metabolites, which have been isolated and identified from the fruiting-body, cultured mycelium and cultured broth of mushrooms [1]*.* Exopolysaccharides (EPS) are referred to secondary metabolite of microorganisms during the growth process, and secreted into the extracellular broth. Recently, many polysaccharides have been isolated from mushrooms [2]. They have emerged as an important class of bioactive natural products in the biochemical and medical areas due to their specific biological activities such as hepatoprotective, antioxidant activity, immunomodulatory property, inhibition early stages of biofouling and gastroprotective effects [3]. The documented immune-active polysaccharides, isolated from *Opuntia polyacantha, Bupleurum smithii,* and *Sipunculus nudus,* exhibited innate immunomodulatory activities through enhancing phagocytosis of macrophages, increasing production of NO and secretion of cytokine [4-6]. Previous reports have demonstrated that EPS from *longan pulp,* and *Enteromorpha prolifera* may not only modulate macrophages activities, but also enhance the spleen lymphocyte proliferation and cytokine production [7,8]. These suggested that EPS represent a potential therapeutics with immunomodulatory action for their low toxicity and high potency.

*Morchella conica,* is an edible mushroom belonging to genus *Morchella*. It was used in Traditional Chinese Medicine to treat indigestion, excessive phlegm and shortness of breath. It has also been consumed as tasted food, health nutritional supplement for its high gastronomic quality, fatigue resistance and gastroprotective effects [9,10]. However, wild *Morchella conica* is difficult to culture, and its price is expensive price [11]. The fermentation broth of *M. conica* contains similar bioactive compounds and is rich in quantity compared with fermentation broth of *M. conica* and wild *M. conica*. Thus, bioactive compounds are obtained from fermentation broth of *M. conica*, and may serve as an ideal substitute for the wild *M. conica*, thereby resolving the problem of its limited production from wilds. To the best of our knowledge, there is no available report yet on the exopolysaccharides from fermentation broth of *Morchella conica*. In the present study, an immunomodulatory activity exopolysaccharide was isolated from submerged fermentation broth of *Morchella conica*. Beside, chemical structure of the isolated polysaccharide was also elucidated.

## Material and method

### Chemicals and materials

The strain of *Morchella conica* (labeled, No. 20110802) was obtained from the Sichuan Agricultural Academy of Sciences, Chengdu, China. DEAE-cellulose 52 and Sephacryl HR-300 were purchased from GE Healthcare Life Sciences (Uppsala, Sweden). Dextran T-2000, T-500, T-200 and T-70 were purchased from Pharmacia Co., Ltd. (Uppsala, Sweden). RAW264.7 cells were obtained from American Type Culture Collection (Manassass, VA, USA). Kunming mice (18~20 g) were obtained from the Animal Facility of the Institute of Chinese Traditional Medicine, Sichuan, China. The protocols of feeding were formed in accordance with the Guidelines of Institute of Chinese Traditional Medicine Animals Research Committee. All other reagents used were of analytical grade.

### Extraction and isolation of exopolysaccharide

*M. conica* was cultured on synthetic potato dextrose agar (PDA) plates in a Petri dish at 25°C for 7 d. Then the seeds were grown in 250 ml Erlenmeyer flasks containing 100 ml liquid culture medium under agitation at 150 rev min^-1^ for 7 days at 25°C. 10% (v/v) of the seed culture was transferred into 20 L culture media (pH 6.4) on a rotary shaker incubator under agitation at 50 rev min^-1^ for 5 d at 28°C. The liquid culture medium used in this study is composed of 3 g/l glucose, 7 g/l sucrose, 3 g/l yeast extract, 5 g/l peptone, 0.1 g/l K_2_HPO_4_, 0.5 g/l KH_2_PO_4_, and 0.5 g/l MgSO_4_. The fermentation broth was harvested and centrifuged at 3000 rpm for 20 min from the culture. After centrifuging, the supernatant was treated by the Sevag method to remove protein and lipid, etc. [12]. In brief, a mixed solution of chloroform: butanol = 4:1 was added in amount of 10% to the supernatant, which was stirred for 12 h. Then, this solution was subjected to centrifugal separation for 20 min at 10000 rpm to remove precipitation, and the supernatant was collected for next study. Next, after adding four-fold volume of 95% ethanol to this supernatant, the crude polysaccharide was obtained through precipitation. This agglutinant was filtered off to remove ethanol, and dialyzed for 12 h (MWCO 5000, Sigma) and lyophilized using Thermo Savant MODULYO freeze-dryer (EC Apparatus Corp., Holbrook, NY, USA) for further study. The sample was re-dissolved in 20 mM Tris–HCl (pH 8.0) and applied to DEAE-Cellulose 52 anion-exchange chromatography column (2.6 × 50 cm, Whatmann) equilibrated with 20 mM Tris–HCl (pH 8.0), and eluted with a linear gradient of NaCl from 0 mM to 500 mM in 20 mM Tris–HCl (pH 8.0) at a flow rate of 2 mL/min. The eluted samples were monitored by the phenol-sulfuric acid method [13], and the four yielded fractions were collected and combined. Subsequently, the samples were further subjected to Sephacryl S-300HR column (2.6 × 100 cm, Pharmacia Co.) and eluted with 10 mM NaCl at a flow rate of 1 mL/min. Fractions containing EPS were pooled and dialyzed. Then, the sample was subjected to lyophilized, and used directly for analysis or stored at -20°C for further study.

### Innate and adaptive immunomodulatory activity of MCP

#### Measurement of NO production

RAW264.7 cells were plated in 24-well culture plates in RPMI 1640 at a density of 1 × 10^6^ for 2 h. LPS (10 μg/ml), and different concentrations MCP (0, 50, 100, 200, 300, 400 and 500 μg/ml) were added, and the mixture was incubated for 48 h. Production of nitric oxide (NO) was measured according to the Griess method [14]. Briefly, supernatants were mixed with an equal volume of Griess reagent, which was prepared by mixing one part of 0.1% (w/v) N-(1-naphthyl) ethylenediamine with one part of 1% (w/v) sulfanilamide in 5% phosphoric acid. After 20 min, absorbance was measured at 540 nm using a UV/vis spectrophotometer (TU-1901, Purkinje General, Beijing, China). The nitrite concentration was calculated using sodium nitrite as a standard.

#### Lymphocyte proliferation test in vitro

Spleen lymphocytes were prepared as reported previously with some modifications [15]. Briefly, mice were killed by cervical dislocation, and obtained spleen sterile. Medium containing spleen lymphocytes was collected, and added equal volume 0.83% Tris-NH_4_Cl for lysis the red blood cells for 5 min. The suspended cells were centrifuged at 1500 rpm for 15 min, and resuspended with complete RPMI-1640. The resuspended cells were subsequently seeded onto culture plates for 1 h at 37°C and 5% CO_2_ to remove the adherent cells. The non-adherent cells were designated as spleen lymphocytes. Cell viability was ≥95% in all experiments. The non-adherent cells, which were spleen lymphocytes, were plated in 96-well culture plates in RPMI 1640 at a density of 1 × 10^6^. Different concentration MCP (0, 50, 100, 200, 300, 400 and 500 μg/ml) was added into 9-wells, and one of three wells added 5 μg/ml ConA, and the other three wells added 5 μg/ml LPS, the rest three wells was just MCP without ConA and LPS. The mixture was incubated for 72 h at 37°C and 5% CO_2_. The splenocytes proliferation was assessed by using MTT-based colorimetric assay.

#### Measurement of homogeneity and molecular weight of MCP

The homogeneity and average molecular weight of MCP were measured and determined by the high performance gel permeation chromatography (HPGPC)method on a Waters 1525 HPLC system (Waters, Boston, US) equipped with a TSK gel 5000 PWXL column (7.8 × 300 mm, Tosoh Co., Tokyo, Japan) and a Alltech 2000ES (Alltech Associates, Inc., USA) Evaporative Light Scatttering Detector (ELSD). The molecular weight was estimated by reference to the calibration curve made under the conditions described above from Dextran T-series standards (T70, T200, T500 and T2000) of known molecular weight.

#### Monosaccharide composition analysis of MCP

The polysaccharide, MCP (~2 mg), was hydrolysed with 2 M trifluoroacetic acid (TFA) for 10 h at 110°C in a sealed glass tube. When the residual acid was removed using methyl alcohol, and the hydrolysate was analyzed by HPLC/ELSD system. The chromatograph was fitted with an Alltech Prevail carbohydrate column (Alltech Associates, Inc., USA). Results were compared with the following monosaccharide standards: D-glucose, L-rhamnose, D-xylose, D-galactose, D-mannose and L-arabinose.

#### Analysis of Infrared (IR) spectra and Nuclear magnetic resonance (NMR)

IR spectroscopy, and ^1^H and ^13^C NMR were used to analyze the structural features of MCP. IR spectrum of MCP were recorded on a Fourier transform infrared spectroscopy (Nicolet 170 SX, FTIR, US), and the test specimens of polysaccharide film were prepared by KBr-disk method. The ^1^H and ^13^C NMR measurements were carried out at 600 and 150 Hz, respectively, on a Bruke 600 Hz NMR instrument (Bruker Avance, Karlsruhe, Germany). All chemical shifts were in relative to Me_4_Si.

#### Statistical analysis

Data were expressed as means ± SD. The significance of difference was evaluated with one-way ANOVA, followed by Student’s *t*-test to statistically identify differences between the control and treated groups. Significant differences were set at *P* < 0.05 and *P* < 0.01.

## Results and discussion

### Isolation and purification of polysaccharide

**Figure 1:**
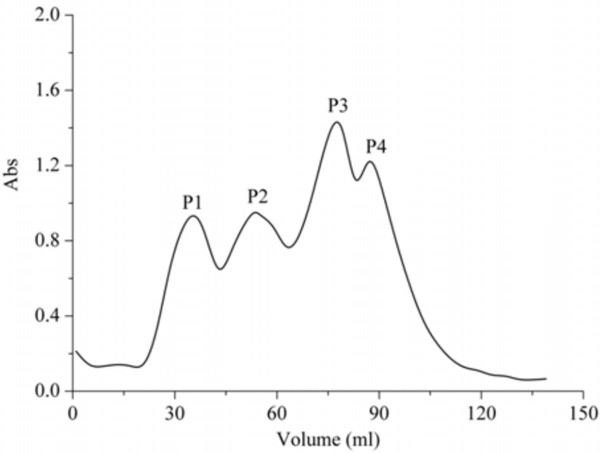
**Elution pattern of the crude polysaccharide produced from submerged mycelial culture of *M.conica *on DEAE-Cellulose 52 anion-exchange chromatography column (2.6 × 50 cm, Whatmann). **The four peaks of P1, P2, P3 and P4 were eluted with a linear gradient of NaCl from 0 mM to 500 mM in 20 mM Tris–HCl (pH 8.0), and the activity were also measured.

During EPS isolation and purification, removing abundant protein, DNA and RNA contaminations in *Morchella conica* extract was an essential step when obtaining EPS. Different methods obtained from the literature were tested to efficiently isolate the soluble EPS fraction from harvested cells [1]. These contaminations were effectively removed by precipitating with 95% ethanol, dialyzed, DEAE-Cellulose 52 anion-exchange (Figure 1) and Sephacryl S-300HR chromatography (data not shown) during the purification procedure. Several sample fractions were taken after each treatment and the relative purification was determined using Nanodrop (DNA and protein) quantification, protein assays and a total carbohydrate assay. As a result, a peak of P3 represents the purified EPS with the purity up to 95%, which designed as MCP. Thus, this process may isolate approximately 539.2 mg EPS from one liter fermentation broth of *M. conica* (Table 1).

**Table 1:** Purification and production yields of Morchella conica polysaccharide

**Steps**	**Volume (ml)**	**Concentration (mg/ml)**	**Total Polysaccharide (mg)**	**Yield (%)**
Crude extract	1000	1.06	10600	100
Pre-treatment	150.3	45.9	6898.8	65.1
DEAE-cellulose	17.4	38.5	669.9	6.32
Sephacryl S-300	26.3	20.5	539.2	5.09

Total polysaccharide is equal to Volume × Concentration. Data represent mean values ± SD (n =3).

### Molecular weight and monosaccharide composition

Molecular weight has been recognized as a critical parameter in the antigenicity of polysaccharides. Most polysaccharides with medicinal properties are high molecules above 100 kDa of molecular weight. Moreover, some polysaccharides have low molecular weights, such as polysaccharide from *Ganoderma lucidum* (8 kDa, 22 kDa), *Euphorbia fischeriana* (49.5 kDa) *and Armillariella tabescens* (49.5 kDa), etc., which were found to exhibit bioactivity [14,16,17]. The molecular weight of purified MCP was about 81.2 kDa by HPLC analysis using dextrans as standards, which was in the range reported for other mushroom polysaccharides. According to HPLC equipped with a Alltech Prevail carbohydrate column and ELSD analyzing, it was found that MCP was composed of only one monosaccharide, D-mannose (data not shown).

### Structure elucidation of MCP

**Figure 2:**
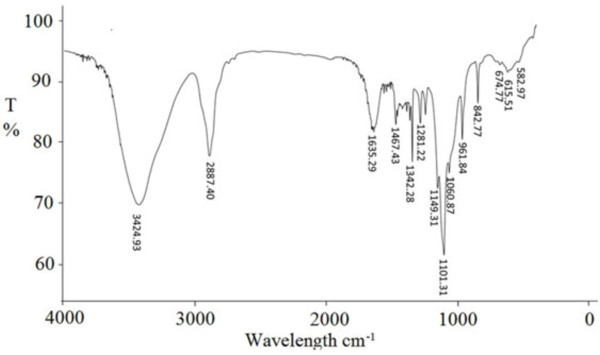
**IR analysis of *M. conica *polysaccharides (MCP).**

**Figure 3:**
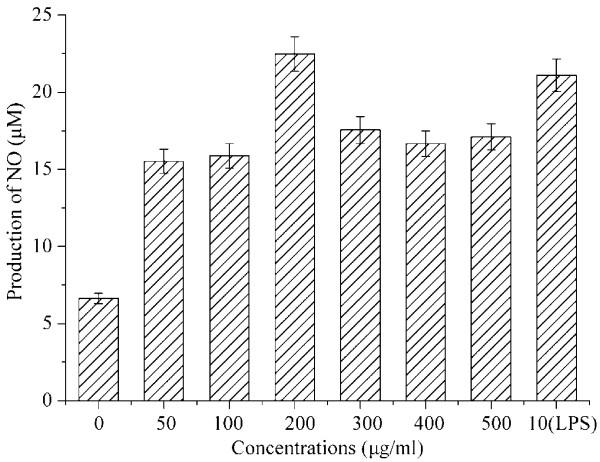
**Effects of polysaccharides of MCP and LPS on NO production in RAW264.7 cells.** Data represent mean values ± SD (n = 3).

It well known that the configuration of polysaccharides was very important for the biological activity. Most of documented immuno-active polysaccharides from medicinal fungi are β-glycosidic linkage polysaccharides [4,16,18]. Recent reports suggested that polysaccharides with α-glycosidic linkage exhibit immune activity from *Ganoderma lucidum, Armillariella tabescens,* and *Cordyceps sinensis*[15,17,19]. As shown in Figure 2, the intensity of bands around 3424.93 cm^−1^ in the IR spectrum was due to the -OH stretching vibration of the polysaccharide and as expected they were broad. The bands in the region of 2887.40 cm^−1^ were due to C-H stretching vibration, and the bands in the region of 1635.29 cm^−1^ were due to associated water. Three strong absorption bands at 1060.87 cm^−1^, 1101.31 cm^−1^ and 1149.31 cm^-1^ in the range of 1200-1000 cm^-1^ in the IR spectrum suggested that the monosaccharide in MCP had a pyranose-ring. The absorption at 842.77 cm^−1^ indicated that MCP had α-glucopyranose linkages. These findings suggested that the MCP have α-glycosidic linkage in the molecule configuration. As shown in Figure 3, the resonances in the region of 98-106 ppm in the ^13^C NMR spectrum of MCP were attributed to the anomeric carbon atoms of mannopyranose (Man*p*), which was in good agreement with the monosaccharide composition [20]. Bases on the above results, it could be concluded that MCP was α-mannopyranose, composed of a repeating unit with the possible structure as [→6)-α-D-Man*p*-(1→6)-[α-D-Man*p*-(1→6)-]n-α-D-Man*p*-(1→.

### Measurement of NO production in vitro

After RAW264.7 cells were incubated with different concentrations of polysaccharides (0, 50, 100, 200, 300, 400 and 500 μg/ml) or LPS (10 μg/ml) for 24 h, NO concentrations in culture supernatants were assessed by NO_2_^-^ contents in the Griess reaction. As shown in Figure 3, 10 μg/ml LPS could significantly increase production of NO (p<0.01), and MCP may significantly induce the production of NO with a dose-dependent manner at 50-200 μg/ml. However, the stimulate roles showed decrease treads when the concentrations of EPS concentrations are higher than 200 μg/ml. Earlier studies showed that the mannose receptor (MR) was existed on the cell surface of macrophage, and involved in the process of production reactive oxidants, phagocytosis, and endocytosis [21]. The polysaccharides with mannose, trehalose and N-acetyl glucosamine residues may bind to MR, and then the complex may active macrophage *via* NF-κB pathway or other signaling pathways, which will enhance the secretion of cytokines, such as IL-1β, IL-6, and GM-CSF, etc. [22,23]. Thus, they play critical role in cell-mediated immunity and humoral immunity. In the present study, results showed that MCP may induce the production of NO, and modulate the innate immune response at the specific concentration ranges.

### Lymphocyte proliferation in vitro

**Figure 4:**
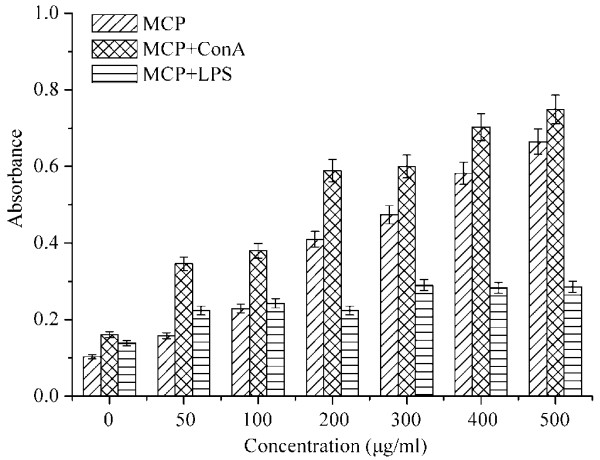
**Effects of different concentrations of MCP, Con A- and LPS-stimulated splenocyte proliferation *in vitro*. **Data represent mean values ± SD (n = 3).

Lymphocyte proliferation is a crucial event in the activation cascade of both cellular and humoral immune responses [3,24]. To investigate the immunomodulatory effect of the polysaccharides, MTT assay was used to evaluate spleen lymphocyte proliferation (Figure 4). The results showed that MCP may promote lymphocyte proliferation in dose-dependent manner. These results are similar with the additions of LPS and/or ConA on lymphocyte proliferation (p<0.05). At a lower concentration of 50 μg/ml, MCP could significantly enhance the proliferation of lymphocyte (P<0.05), and the MCP concentration of 500 μg/ml exhibited the highest co-mitogenic activities compared to those of the normal control groups. It is well known that lymphocyte proliferation effect were either directly activated or by the cytokines exopolysaccharide-induced by other cells secretion such as macrophages and natural killer (NK). Earlier reports suggested that polysaccharides from *Morchella esculenta* could directly activate T cells [24]. Moreover, ConA also can selectively promote the proliferation of T cells. Thus, the effects of MCP on lymphocyte proliferation may be an adaptive immune response *via* T cell-mediated. However, the mechanism of MCP is directly activated *via* T cells or by the cytokines need further study.

## Conclusion

Culture of *M. conica* fruiting bodies usually takes at least three months; therefore compared with the extraction of polysaccharides from the fruiting bodies of *M. conica,* the production and purification of polysaccharides from fermentation broth can significantly shorten the culture period and provide a faster way of polysaccharide production. In the present study, we showed, for the first time that the polysaccharides isolated and characterized from fermentation broth of *M. conica*. In addition, it is important to note that MCP has immunomodulatory activity, and may be seen as a promising immunopotentiating agent in health-care food or the treatment of infectious diseases. Further in-depth study will focus on between the mechanism of immunomodulatory activity and structure-function relationship.

## Competing interests

The authors declare that they have no competing interests.

## Authors’ contribution

CS, Experiment operator; XX, data collection and analysis; DL, literature search; MW, Manuscript editing and review; FZ, Manuscript editing and review; MY, fermentation culture assistant; WW, fermentation culture assistant; NJ, figures preparation; LX, Experiment design and manuscript writing. All authors read and approved the final manuscript.
